# (*E*)-2-Hy­droxy-4-meth­oxy-3-(3-methyl­but-2-en­yl)-6-styryl­benzoic acid

**DOI:** 10.1107/S1600536812050258

**Published:** 2012-12-15

**Authors:** Xingyue Ji, Jie Jin, Guanghui Zheng, Zhuorong Li

**Affiliations:** a Institute of Medicinal Biotechnology, Chinese Academy of Medical Sciences and Peking Union Medical College, Beijing 100050, People’s Republic of China

## Abstract

The title compound, C_21_H_22_O_4_, also known as cajanine, features an intra­molecular O—H⋯O hydrogen bond between the adjacent carb­oxy and hy­droxy groups. The benzene rings make an inter­planar angle of 175.4 (2)°. In the crystal, mol­ecules are linked by pairs of O—H⋯O hydrogen bonds, forming inversion dimers.

## Related literature

Cajanine is an important component of the herb *Cajanus cajan* L., which is used in traditional Chinese medicine to treat osteonecrosis of the femoral head. For the total synthesis of cajanine, see: Ji *et al.* (2011 ▶). For the bioactivity of cajanine, see: Fu *et al.* (2009 ▶); Ji *et al.* (2011 ▶); Luo *et al.* (2008*a* ▶,*b* ▶); Zheng *et al.* (2007*a* ▶,*b* ▶); Inman & Hopp (2002 ▶); Ruan *et al.* (2009 ▶).
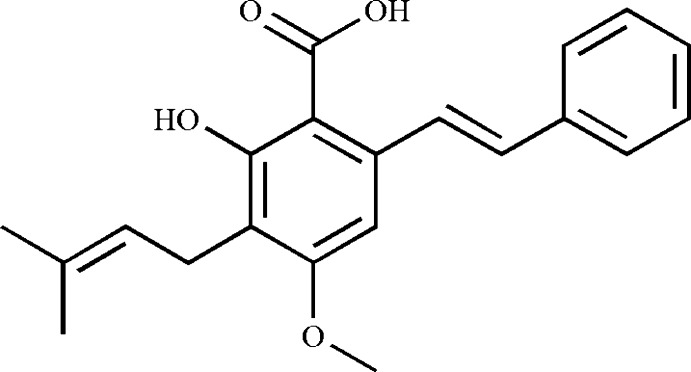


## Experimental

### 

#### Crystal data

C_21_H_22_O_4_
*M* *_r_* = 338.39
Triclinic, 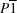

*a* = 6.9790 (3) Å
*b* = 9.9975 (8) Å
*c* = 13.8202 (11) Å
α = 77.899 (1)°
β = 78.956 (2)°
γ = 78.507 (2)°
*V* = 912.58 (11) Å^3^
*Z* = 2
Mo *K*α radiation
μ = 0.08 mm^−1^
*T* = 298 K
0.35 × 0.32 × 0.31 mm

#### Data collection

Bruker SMART APEX diffractometer
4547 measured reflections
3138 independent reflections
1735 reflections with *I* > 2σ(*I*)
*R*_int_ = 0.024

#### Refinement

*R*[*F* ^2^ > 2σ(*F* ^2^)] = 0.052
*wR*(*F* ^2^) = 0.152
*S* = 1.06
3138 reflections
229 parameters
H-atom parameters constrained
Δρ_max_ = 0.15 e Å^−3^
Δρ_min_ = −0.18 e Å^−3^

### 

Data collection: *SMART* (Bruker, 1999 ▶); cell refinement: *SAINT* (Bruker, 1999 ▶); data reduction: *SAINT*; program(s) used to solve structure: *SHELXS97* (Sheldrick, 2008 ▶); program(s) used to refine structure: *SHELXL97* (Sheldrick, 2008 ▶); molecular graphics: *SHELXTL* (Sheldrick 2008 ▶); software used to prepare material for publication: *SHELXTL*.

## Supplementary Material

Crystal structure: contains datablock(s) I, global. DOI: 10.1107/S1600536812050258/ng5309sup1.cif

Structure factors: contains datablock(s) I. DOI: 10.1107/S1600536812050258/ng5309Isup2.hkl

Supplementary material file. DOI: 10.1107/S1600536812050258/ng5309Isup3.cml

Additional supplementary materials:  crystallographic information; 3D view; checkCIF report

## Figures and Tables

**Table 1:** Hydrogen-bond geometry (Å, °)

*D*—H⋯*A*	*D*—H	H⋯*A*	*D*⋯*A*	*D*—H⋯*A*
O1—H1⋯O2^i^	0.82	1.85	2.667 (2)	176
O3—H3⋯O2	0.82	1.82	2.546 (2)	147

Symmetry code: (i) 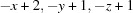.

**Fig. 1.**
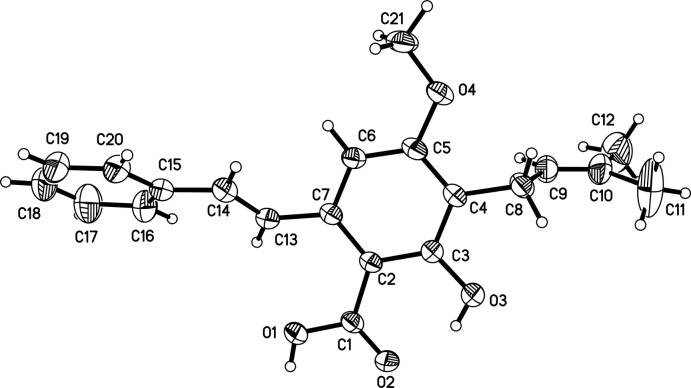
The title molecule with the atom-numbering scheme. Ddisplacement parameters are shown at the 30% probability level.

**Fig. 2.**
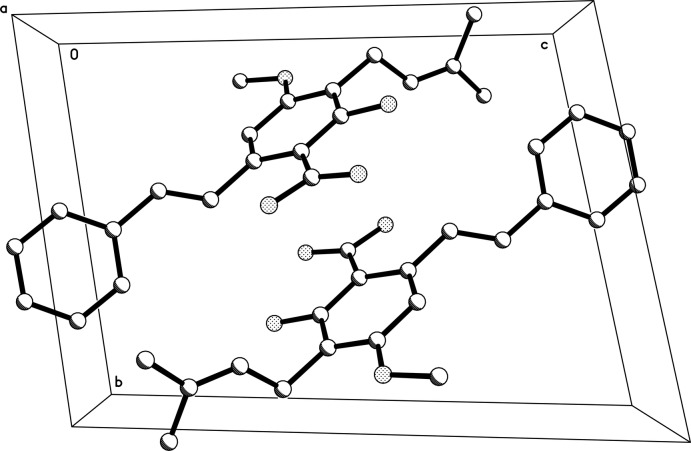
Packing of the title molecules viewed along the a direction.

## supplementary crystallographic information

### Comment

Cajanine, also known as longistylineA-2-carboxylicacid, is a stilbene derivative isolated from the herb Cajanus cajan *L*. The herb has been used in traditional Chinese medicine for many years to treat osteonecrosis of the femoralhead. Cajanine is a good drug candidate because of its wide range of pharmacological activities, which include antitumor (Ji *et al.* 2011), anti- HSV (Fu *et al.* 2009), anti-hyperlipidemic (Luo *et al.*, 2008*a*), anti-osteoporotic (Zheng *et al.*, 2007*a*,*b*), hypoglycemic (Inman & Hopp, 2002) and antioxidant (Luo *et al.*, 2008*b*) effects. It was also reported that cajanine can modulate A*β*_25–35_-induced cognitive deficits, oxidative stress and cholinergic dysfunction in mice (Ruan *et al.*, 2009). We have accomplished its total synthesis previously (Ji *et al.* 2011), and the bioassay results showed that it showed some antiproliferative activity against human hepatoma cells.

The crystal structure of the title compound is reported here. In this crystal, the two benzene rings are not in the same plane, and the interplanar angle between them is 175.4 (2) °. A strong intramolecular O—H···O hydrogen bond is formed between the carboxyl group and a hydroxy group.

### Experimental

(*E*)-Methyl 2-hydroxy-4-methoxy-3-(3-methylbut-2-enyl)-6-styrylbenzoate (0.5 g, 1.42 mmol) was dissolved in EtOH/H_2_O (15 ml/5 ml) and KOH (0.25 g, 4.26 mmol) was added. The mixture was heated under reflux for 3 h, and the reaction mixture was then added into ice water (50 ml) and the pH was adjusted to 2 with 10% HCl. The precipitate obtained was filtered and washed with water, dried in vacuum. The crude product was recrystallized from ethyl acetate / petroleum ether to give colorless crystals (0.38 g, 80%). m.p.168–170 °C.

^1^H NMR(400 MHz,CDCl_3_, 25 °C, TMS) δ: 11.58(s, 1H), 7.81(d, *J* = 16.0 Hz, 1H), 7.52(d, *J* = 7.2 Hz, 2H),7.38(t, *J* = 7.2 Hz,2*H*), 7.28(t, *J* = 7.2 Hz, 1H), 6.83(d, *J* = 16.0 Hz, 1H), 6.65(s, 1H), 5.22(t, *J* = 6.8 Hz, 1H), 3.95(s, 3H), 3.38(d, *J* = 6.8 Hz, 2H), 1.79(s, 3H), 1.68 (s, 3H), COOH was not observed. ^13^C NMR(100 MHz, CDCl_3_) δ: 174.95, 162.44, 162.25, 141.77, 137.28, 131.97, 130.86, 130.34, 128.74, 127.89, 126.79, 121.89, 116.77, 103.29, 102.97, 55.73, 25.82, 22.09, 17.80. HRMS(ESI) calcd for C_21_H_22_O_4_Na [*M*+Na]^+^ 361.14158, found 361.14318.

### Refinement

Hydrogens were generated geometrically.

### Crystal data

**Table:**

C_21_H_22_O_4_	*Z* = 2
*M**_r_* = 338.39	*F*(000) = 360
Triclinic, *P*1	*D*_x_ = 1.231 Mg m^−^^3^
*a* = 6.9790 (3) Å	Mo *K*α radiation, λ = 0.71073 Å
*b* = 9.9975 (8) Å	Cell parameters from 1203 reflections
*c* = 13.8202 (11) Å	θ = 2.8–25.6°
α = 77.899 (1)°	µ = 0.08 mm^−^^1^
β = 78.956 (2)°	*T* = 298 K
γ = 78.507 (2)°	Lump, colorless
*V* = 912.58 (11) Å^3^	0.35 × 0.32 × 0.31 mm

### Data collection

**Table:**

Bruker SMART APEX diffractometer	1735 reflections with *I* > 2σ(*I*)
Radiation source: fine-focus sealed tube	*R*_int_ = 0.024
Graphite monochromator	θ_max_ = 25.0°, θ_min_ = 3.0°
ω scans	*h* = −8→8
4547 measured reflections	*k* = −11→11
3138 independent reflections	*l* = −16→12

### Refinement

**Table:**

Refinement on *F*^2^	Primary atom site location: structure-invariant direct methods
Least-squares matrix: full	Secondary atom site location: difference Fourier map
*R*[*F*^2^ > 2σ(*F*^2^)] = 0.052	Hydrogen site location: inferred from neighbouring sites
*wR*(*F*^2^) = 0.152	H-atom parameters constrained
*S* = 1.06	*w* = 1/[σ^2^(*F*_o_^2^) + (0.057*P*)^2^ + 0.0883*P*] where *P* = (*F*_o_^2^ + 2*F*_c_^2^)/3
3138 reflections	(Δ/σ)_max_ = 0.001
229 parameters	Δρ_max_ = 0.15 e Å^−^^3^
0 restraints	Δρ_min_ = −0.18 e Å^−^^3^

### Special details

**Table:**

Geometry. All e.s.d.'s (except the e.s.d. in the dihedral angle between two l.s. planes) are estimated using the full covariance matrix. The cell e.s.d.'s are taken into account individually in the estimation of e.s.d.'s in distances, angles and torsion angles; correlations between e.s.d.'s in cell parameters are only used when they are defined by crystal symmetry. An approximate (isotropic) treatment of cell e.s.d.'s is used for estimating e.s.d.'s involving l.s. planes.
Refinement. Refinement of *F*^2^ against ALL reflections. The weighted *R*-factor *wR* and goodness of fit *S* are based on *F*^2^, conventional *R*-factors *R* are based on *F*, with *F* set to zero for negative *F*^2^. The threshold expression of *F*^2^ > σ(*F*^2^) is used only for calculating *R*-factors(gt) *etc*. and is not relevant to the choice of reflections for refinement. *R*-factors based on *F*^2^ are statistically about twice as large as those based on *F*, and *R*- factors based on ALL data will be even larger.

### Fractional atomic coordinates and isotropic or equivalent isotropic displacement parameters (Å^2^)

**Table:**

	*x*	*y*	*z*	*U*_iso_*/*U*_eq_	
O1	0.8842 (3)	0.47521 (18)	0.39744 (12)	0.0647 (5)	
H1	0.9635	0.5150	0.4123	0.097*	
O2	0.8460 (3)	0.39954 (17)	0.56073 (13)	0.0578 (5)	
O3	0.6238 (3)	0.21943 (17)	0.64005 (12)	0.0622 (5)	
H3	0.7100	0.2662	0.6362	0.093*	
O4	0.1871 (3)	0.12244 (19)	0.45984 (13)	0.0699 (6)	
C1	0.7933 (3)	0.4066 (2)	0.47912 (19)	0.0460 (6)	
C2	0.6332 (3)	0.3384 (2)	0.46785 (17)	0.0425 (6)	
C3	0.5566 (3)	0.2451 (2)	0.55136 (17)	0.0460 (6)	
C4	0.4102 (3)	0.1710 (2)	0.54695 (18)	0.0474 (6)	
C5	0.3353 (4)	0.1946 (3)	0.45837 (19)	0.0508 (7)	
C6	0.4050 (4)	0.2864 (2)	0.37512 (18)	0.0500 (6)	
H6	0.3517	0.2992	0.3165	0.060*	
C7	0.5533 (3)	0.3596 (2)	0.37766 (17)	0.0442 (6)	
C8	0.3312 (4)	0.0732 (3)	0.63820 (18)	0.0568 (7)	
H8A	0.2964	−0.0038	0.6164	0.068*	
H8B	0.4356	0.0358	0.6786	0.068*	
C9	0.1559 (4)	0.1381 (3)	0.7015 (2)	0.0639 (8)	
H9	0.0923	0.2237	0.6723	0.077*	
C10	0.0802 (5)	0.0907 (3)	0.7923 (2)	0.0786 (9)	
C11	0.1714 (8)	−0.0452 (5)	0.8471 (3)	0.170 (2)	
H11A	0.1457	−0.1193	0.8198	0.255*	
H11B	0.1155	−0.0549	0.9168	0.255*	
H11C	0.3118	−0.0485	0.8400	0.255*	
C12	−0.0985 (6)	0.1646 (4)	0.8487 (3)	0.1287 (15)	
H12A	−0.1369	0.2547	0.8104	0.193*	
H12B	−0.0693	0.1745	0.9117	0.193*	
H12C	−0.2048	0.1124	0.8603	0.193*	
C13	0.6113 (4)	0.4597 (3)	0.28730 (18)	0.0516 (7)	
H13	0.6350	0.5444	0.2960	0.062*	
C14	0.6320 (4)	0.4374 (3)	0.1946 (2)	0.0628 (8)	
H14	0.6173	0.3498	0.1874	0.075*	
C15	0.6759 (4)	0.5363 (4)	0.1019 (2)	0.0683 (8)	
C16	0.6483 (5)	0.6766 (4)	0.1000 (2)	0.0940 (11)	
H16	0.6044	0.7111	0.1593	0.113*	
C17	0.6849 (7)	0.7665 (5)	0.0111 (3)	0.1376 (17)	
H17	0.6664	0.8613	0.0107	0.165*	
C18	0.7484 (8)	0.7173 (7)	−0.0768 (4)	0.139 (2)	
H18	0.7703	0.7791	−0.1367	0.167*	
C19	0.7796 (6)	0.5793 (7)	−0.0771 (3)	0.1203 (17)	
H19	0.8273	0.5457	−0.1366	0.144*	
C20	0.7398 (5)	0.4888 (4)	0.0121 (2)	0.0903 (11)	
H20	0.7564	0.3943	0.0116	0.108*	
C21	0.1162 (4)	0.1248 (3)	0.3689 (2)	0.0719 (8)	
H21A	0.0509	0.2167	0.3455	0.108*	
H21B	0.0245	0.0610	0.3810	0.108*	
H21C	0.2257	0.0983	0.3191	0.108*	

### Atomic displacement parameters (Å^2^)

**Table:**

	*U*^11^	*U*^22^	*U*^33^	*U*^12^	*U*^13^	*U*^23^
O1	0.0679 (12)	0.0791 (13)	0.0572 (11)	−0.0449 (10)	−0.0144 (9)	0.0008 (10)
O2	0.0604 (12)	0.0644 (12)	0.0567 (11)	−0.0276 (9)	−0.0195 (9)	−0.0032 (9)
O3	0.0674 (12)	0.0704 (12)	0.0540 (11)	−0.0300 (10)	−0.0169 (9)	0.0021 (9)
O4	0.0663 (13)	0.0852 (14)	0.0689 (13)	−0.0438 (11)	−0.0076 (10)	−0.0106 (10)
C1	0.0412 (15)	0.0437 (15)	0.0537 (16)	−0.0126 (12)	−0.0063 (13)	−0.0060 (12)
C2	0.0386 (14)	0.0433 (14)	0.0484 (14)	−0.0123 (11)	−0.0041 (11)	−0.0114 (11)
C3	0.0459 (15)	0.0477 (15)	0.0452 (14)	−0.0110 (12)	−0.0071 (12)	−0.0068 (12)
C4	0.0431 (15)	0.0463 (15)	0.0539 (15)	−0.0160 (12)	−0.0011 (12)	−0.0089 (12)
C5	0.0436 (15)	0.0542 (16)	0.0600 (16)	−0.0195 (13)	−0.0015 (13)	−0.0171 (13)
C6	0.0474 (15)	0.0564 (16)	0.0513 (15)	−0.0165 (13)	−0.0098 (12)	−0.0119 (13)
C7	0.0400 (14)	0.0453 (14)	0.0491 (14)	−0.0129 (11)	−0.0039 (11)	−0.0103 (12)
C8	0.0527 (17)	0.0517 (16)	0.0648 (17)	−0.0180 (13)	−0.0067 (14)	−0.0014 (13)
C9	0.0547 (18)	0.0699 (19)	0.0581 (18)	−0.0097 (15)	−0.0068 (14)	0.0062 (15)
C10	0.085 (2)	0.092 (2)	0.0524 (18)	−0.0209 (19)	−0.0035 (17)	0.0007 (17)
C11	0.214 (5)	0.129 (4)	0.099 (3)	0.012 (4)	0.032 (3)	0.041 (3)
C12	0.112 (3)	0.169 (4)	0.081 (3)	−0.009 (3)	0.022 (2)	−0.016 (3)
C13	0.0487 (16)	0.0574 (16)	0.0534 (16)	−0.0189 (13)	−0.0129 (12)	−0.0063 (13)
C14	0.0596 (18)	0.075 (2)	0.0587 (18)	−0.0266 (15)	−0.0070 (14)	−0.0099 (15)
C15	0.0539 (18)	0.102 (3)	0.0540 (18)	−0.0332 (17)	−0.0130 (14)	−0.0010 (17)
C16	0.110 (3)	0.101 (3)	0.068 (2)	−0.039 (2)	−0.0156 (19)	0.015 (2)
C17	0.161 (4)	0.140 (4)	0.099 (3)	−0.062 (3)	−0.025 (3)	0.045 (3)
C18	0.124 (4)	0.208 (6)	0.077 (3)	−0.080 (4)	−0.030 (3)	0.055 (4)
C19	0.085 (3)	0.221 (5)	0.055 (2)	−0.051 (4)	−0.0123 (19)	0.001 (3)
C20	0.075 (2)	0.145 (3)	0.056 (2)	−0.038 (2)	−0.0091 (16)	−0.014 (2)
C21	0.0644 (19)	0.083 (2)	0.085 (2)	−0.0313 (16)	−0.0194 (16)	−0.0250 (17)

### Geometric parameters (Å, º)

**Table:**

O1—C1	1.312 (3)	C11—H11A	0.9600
O1—H1	0.8200	C11—H11B	0.9600
O2—C1	1.236 (3)	C11—H11C	0.9600
O3—C3	1.352 (3)	C12—H12A	0.9600
O3—H3	0.8200	C12—H12B	0.9600
O4—C5	1.368 (3)	C12—H12C	0.9600
O4—C21	1.430 (3)	C13—C14	1.322 (3)
C1—C2	1.467 (3)	C13—H13	0.9300
C2—C3	1.414 (3)	C14—C15	1.467 (4)
C2—C7	1.420 (3)	C14—H14	0.9300
C3—C4	1.393 (3)	C15—C16	1.372 (4)
C4—C5	1.379 (3)	C15—C20	1.384 (4)
C4—C8	1.511 (3)	C16—C17	1.375 (4)
C5—C6	1.387 (3)	C16—H16	0.9300
C6—C7	1.391 (3)	C17—C18	1.367 (6)
C6—H6	0.9300	C17—H17	0.9300
C7—C13	1.472 (3)	C18—C19	1.354 (6)
C8—C9	1.482 (4)	C18—H18	0.9300
C8—H8A	0.9700	C19—C20	1.384 (5)
C8—H8B	0.9700	C19—H19	0.9300
C9—C10	1.294 (4)	C20—H20	0.9300
C9—H9	0.9300	C21—H21A	0.9600
C10—C12	1.487 (4)	C21—H21B	0.9600
C10—C11	1.494 (5)	C21—H21C	0.9600
			
C1—O1—H1	109.5	C10—C11—H11C	109.5
C3—O3—H3	109.5	H11A—C11—H11C	109.5
C5—O4—C21	119.8 (2)	H11B—C11—H11C	109.5
O2—C1—O1	120.0 (2)	C10—C12—H12A	109.5
O2—C1—C2	122.9 (2)	C10—C12—H12B	109.5
O1—C1—C2	117.1 (2)	H12A—C12—H12B	109.5
C3—C2—C7	118.5 (2)	C10—C12—H12C	109.5
C3—C2—C1	117.8 (2)	H12A—C12—H12C	109.5
C7—C2—C1	123.7 (2)	H12B—C12—H12C	109.5
O3—C3—C4	115.6 (2)	C14—C13—C7	124.5 (2)
O3—C3—C2	122.2 (2)	C14—C13—H13	117.8
C4—C3—C2	122.2 (2)	C7—C13—H13	117.8
C5—C4—C3	117.8 (2)	C13—C14—C15	126.9 (3)
C5—C4—C8	121.7 (2)	C13—C14—H14	116.6
C3—C4—C8	120.5 (2)	C15—C14—H14	116.6
O4—C5—C4	115.0 (2)	C16—C15—C20	118.1 (3)
O4—C5—C6	123.2 (2)	C16—C15—C14	122.2 (3)
C4—C5—C6	121.7 (2)	C20—C15—C14	119.6 (3)
C5—C6—C7	121.3 (2)	C15—C16—C17	120.5 (4)
C5—C6—H6	119.4	C15—C16—H16	119.7
C7—C6—H6	119.4	C17—C16—H16	119.7
C6—C7—C2	118.5 (2)	C18—C17—C16	120.4 (5)
C6—C7—C13	117.4 (2)	C18—C17—H17	119.8
C2—C7—C13	124.0 (2)	C16—C17—H17	119.8
C9—C8—C4	114.1 (2)	C19—C18—C17	120.4 (4)
C9—C8—H8A	108.7	C19—C18—H18	119.8
C4—C8—H8A	108.7	C17—C18—H18	119.8
C9—C8—H8B	108.7	C18—C19—C20	119.2 (4)
C4—C8—H8B	108.7	C18—C19—H19	120.4
H8A—C8—H8B	107.6	C20—C19—H19	120.4
C10—C9—C8	128.2 (3)	C19—C20—C15	121.3 (4)
C10—C9—H9	115.9	C19—C20—H20	119.4
C8—C9—H9	115.9	C15—C20—H20	119.4
C9—C10—C12	123.6 (3)	O4—C21—H21A	109.5
C9—C10—C11	120.6 (3)	O4—C21—H21B	109.5
C12—C10—C11	115.8 (3)	H21A—C21—H21B	109.5
C10—C11—H11A	109.5	O4—C21—H21C	109.5
C10—C11—H11B	109.5	H21A—C21—H21C	109.5
H11A—C11—H11B	109.5	H21B—C21—H21C	109.5
			
O2—C1—C2—C3	−9.3 (3)	C3—C2—C7—C6	−0.8 (3)
O1—C1—C2—C3	169.4 (2)	C1—C2—C7—C6	178.3 (2)
O2—C1—C2—C7	171.6 (2)	C3—C2—C7—C13	176.3 (2)
O1—C1—C2—C7	−9.7 (3)	C1—C2—C7—C13	−4.6 (4)
C7—C2—C3—O3	−179.7 (2)	C5—C4—C8—C9	−86.0 (3)
C1—C2—C3—O3	1.1 (3)	C3—C4—C8—C9	91.3 (3)
C7—C2—C3—C4	1.8 (3)	C4—C8—C9—C10	−165.1 (3)
C1—C2—C3—C4	−177.4 (2)	C8—C9—C10—C12	−179.4 (3)
O3—C3—C4—C5	179.4 (2)	C8—C9—C10—C11	0.7 (6)
C2—C3—C4—C5	−2.1 (3)	C6—C7—C13—C14	−41.1 (3)
O3—C3—C4—C8	1.9 (3)	C2—C7—C13—C14	141.8 (3)
C2—C3—C4—C8	−179.5 (2)	C7—C13—C14—C15	175.4 (2)
C21—O4—C5—C4	−171.9 (2)	C13—C14—C15—C16	−18.2 (5)
C21—O4—C5—C6	8.9 (4)	C13—C14—C15—C20	164.4 (3)
C3—C4—C5—O4	−177.8 (2)	C20—C15—C16—C17	−0.5 (5)
C8—C4—C5—O4	−0.4 (3)	C14—C15—C16—C17	−177.9 (3)
C3—C4—C5—C6	1.4 (4)	C15—C16—C17—C18	0.4 (6)
C8—C4—C5—C6	178.8 (2)	C16—C17—C18—C19	−1.3 (8)
O4—C5—C6—C7	178.7 (2)	C17—C18—C19—C20	2.3 (7)
C4—C5—C6—C7	−0.4 (4)	C18—C19—C20—C15	−2.4 (6)
C5—C6—C7—C2	0.1 (3)	C16—C15—C20—C19	1.5 (5)
C5—C6—C7—C13	−177.2 (2)	C14—C15—C20—C19	179.0 (3)

### Hydrogen-bond geometry (Å, º)

**Table:**

*D*—H···*A*	*D*—H	H···*A*	*D*···*A*	*D*—H···*A*
O1—H1···O2^i^	0.82	1.85	2.667 (2)	176
O3—H3···O2	0.82	1.82	2.546 (2)	147

Symmetry code: (i) −*x*+2, −*y*+1, −*z*+1.
